# Regulatory T cells administration reduces anxiety-like behavior in mice submitted to chronic restraint stress

**DOI:** 10.3389/fncel.2024.1406832

**Published:** 2024-08-14

**Authors:** Yamila Cepeda, Roberto Elizondo-Vega, Camila Garrido, Catalina Tobar, Matías Araneda, Patricia Oliveros, Patricio Ordenes, Claudio Carril, Pía M. Vidal, Patricia Luz-Crawford, María. A. García-Robles, Karina Oyarce

**Affiliations:** ^1^Laboratorio de Neuroinmunología, Facultad de Medicina y Ciencia, Universidad San Sebastián, Sede Concepción, Concepción, Chile; ^2^Laboratorio de Biología Celular, Facultad de Ciencias Biológicas, Universidad de Concepción, Concepción, Chile; ^3^Neuroimmunology and Regeneration of the Central Nervous System Unit, Biomedical Science Research Laboratory, Department of Basic Sciences, Faculty of Medicine, Universidad Católica de la Santísima Concepción, Concepción, Chile; ^4^Centro de Investigación e Innovación Biomédica, Facultad de Medicina, Universidad de Los Andes, Santiago, Chile; ^5^IMPACT, Center of Interventional Medicine for Precision and Advanced Cellular Therapy, Santiago, Chile

**Keywords:** depression, anxiety, inflammation, neuroinflammation, regulatory T cells (Treg cells), chronic restraint stress (CRS), chronic unpredictable mild stress (CUS)

## Abstract

**Background:**

Major depression disorder (MDD) and anxiety are common mental disorders that significantly affect the quality of life of those who suffer from them, altering the person’s normal functioning. From the biological perspective, the most classical hypothesis explaining their occurrence relies on neurotransmission and hippocampal excitability alterations. However, around 30% of MDD patients do not respond to medication targeting these processes. Over the last decade, the involvement of inflammatory responses in depression and anxiety pathogenesis has been strongly acknowledged, opening the possibility of tackling these disorders from an immunological point of view. In this context, regulatory T cells (Treg cells), which naturally maintain immune homeostasis by suppressing inflammation could be promising candidates for their therapeutic use in mental disorders.

**Methods:**

To test this hypothesis, C57BL/6 adult male mice were submitted to classical stress protocols to induce depressive and anxiety-like behavior; chronic restriction stress (CRS), and chronic unpredictable stress (CUS). Some of the stressed mice received a single adoptive transfer of Treg cells during stress protocols. Mouse behavior was analyzed through the open field (OFT) and forced swim test (FST). Blood and spleen samples were collected for T cell analysis using cell cytometry, while brains were collected to study changes in microglia by immunohistochemistry.

**Results:**

Mice submitted to CRS and CUS develop anxiety and depressive-like behavior, and only CRS mice exhibit lower frequencies of circulating Treg cells. Adoptive transfer of Treg cells decreased anxiety-like behavior in the OFT only in CRS model, but not depressive behavior in FST in neither of the two models. In CRS mice, Treg cells administration lowered the number of microglia in the hippocampus, which increased due this stress paradigm, and restored its arborization. However, in CUS mice, Treg cells administration increased microglia number with no significant effect on their arborization.

**Conclusion:**

Our results for effector CD4^+^ T cells in the spleen and microglia number and morphology in the hippocampus add new evidence in favor of the participation of inflammatory responses in the development of depressive and anxiety-like behavior and suggest that the modulation of key immune cells such as Treg cells, could have beneficial effects on these disorders.

## Introduction

Depression and anxiety are two common mental disorders that affects ~300 million people worldwide (WHO, 2024). It has been estimated that since the COVID-19 pandemic, the incidence of both disorders has increased (WHO, 2022), and about a third of depressed patients do not respond to the canonical line of treatment, which consists mainly in serotonin reuptake inhibitors (Gaynes et al., 2009). In the last decade, several studies have shown that peripheral low-grade inflammation and neuroinflammation occurs in animal models of depression and at least in some depressed patients, which exhibit higher levels of pro-inflammatory cytokines in serum, such as: interleukin (IL)-4, IL-6, and tumor necrosis factor (TNF)-α (Felger and Lotrich, 2013; Obermanns et al., 2021; Chen et al., 2023), and higher frequencies of pathogenic Th17 proinflammatory cells (Chen et al., 2011; Davami et al., 2016; Beurel and Lowell, 2018). In addition, the use of anti-inflammatory drugs in depressed patients has shown improvement in their symptoms (Köhler et al., 2014; Abbott et al., 2015), suggesting that targeting inflammation could be useful as a complementary treatment.

Regulatory T cells (Treg cells) are a special CD4^+^ T cell subpopulation characterized by the expression of the master gene regulator FOXP3. They are crucial for immune homeostasis because of their ability to control inflammation by suppressing effector T cells proliferation through a vast array of different mechanisms (Dikiy and Rudensky, 2023). In both human and animal models of depression (Grosse et al., 2016a,b), such as postpartum depression in rats (Li et al., 2016), a lower frequency of Treg cells has been detected, correlating with pro-inflammatory phenotype, suggesting that Treg cells impairment might also play a role in either the development or maintenance of depressive behavior. Lately it has been described that IL-2, a potent Treg cells inductor, crucial for their survival, normalized anxiety and depressive-like behavior in a CUS model, also normalizing neurotransmitter concentrations and cytokines IL-17, tumor growth factor (TGF)-β and IL-6 in the hippocampus (Huang et al., 2022). Altogether, this evidence suggests that Treg cells-based therapy could be a promising strategy for anxiety and depression. However, it is important to acknowledge that to date, there is a lack of reports demonstrating the behavioral effects of exogenous Treg cells administration.

In this study, the investigation focused on determining whether Treg cells adoptive transfer could modulate inflammation and decrease anxious and depressive-like behavior in male mice submitted to CRS and CUS. Our findings show that peripheral Treg cells administration decreased anxiety, but not depressive-like behavior only in the CRS model, while also modulating microglia number in the hippocampus dentate gyrus.

## Materials and methods

### Animals

C57BL/6 adult male mice (10–12 weeks old) were used for the experiments. They were maintained in the CREAV facility (Universidad de Concepción, Chile), with a 12 h light/dark cycle, and *ad libitum* access to water and chow diet (Lab Diet, 5P00 Prolab RMH 3000, Purina Mills, St. Louis, MO) under veterinarian supervision throughout the experiment period. Mice were group housed (with a maximum of 5 mice per cage) in polycarbonate cages (13 × 19 × 25 cm), with cob bedding, cardboard cylinders, and paper shreds as nesting material/environment enrichment. Randomly some of the cages were submitted to chronic stress by restriction (CRS) or Chronic unpredictable stress (CUS), and by the end of the experimental protocol, behavior tests were performed. All mice used for experimentation were in good health according to standardized supervision guidelines. After behavior analysis, all mice were euthanized by cervical dislocation under anesthesia with a mixture of ketamine 80 mg/kg and xylazine 10 mg/kg intraperitoneal. Staff that administrated Treg cells or vehicle was different from the staff that processed blood and tissue samples and from the staff that assessed the effects on behavior and cellular analysis. All procedures were performed according to the Chilean National Research Agency (ANID) Guidelines for animal experimentation. This study was approved by the Ethics Committee of Universidad San Sebastian (internal code 03-2019-20) and Universidad de Concepción (internal code CEBB533-2019).

### Chronic restriction stress

For CRS paradigm, mice were gently introduced into 50 mL centrifuge tubes that had ventilation holes and were attached to a cardboard surface, for 6 h daily, for 28 days, as described by Chiba et al. (2012), Jangra et al. (2017), Zhu et al. (2019), and Misztak et al. (2021). During the 6 h period, control mice were also food and water-deprived. In total, 2 independent experiments were conducted to see differences between control mice and mice exposed to CRS, with *n* = 5 and *n* = 6; while 3 independent experiments were conducted to evaluate the effect of Treg cells in CRS mice, with an *n* = 11 for the control group, *n* = 9 for CRS group and *n* = 10 for CRS + Treg cells group.

### Chronic unpredictable stress

For CUS paradigm, mice were exposed to two different uncomfortable stimuli daily for 42 days that were randomly scheduled so mice could not habituate (Table 1). These stimuli are considered mild and included movement restriction for 1 h, shaking at 150 rpm for 1 h, cage tilt at an angle of 45° overnight, 24 h water or food deprivation, warm air stream for 10 min (with pauses every 2 min) and exposure to male rat feces and hair. In total, 2 independent experiments were conducted with an *n* = 9 for the control group, *n* = 9 for CUS group and *n* = 8 for CUS + Treg cells group.

**Table 1:** Example of stress schedule for CUS paradigm.

Monday	Tuesday	Wednesday	Thursday	Friday	Saturday	Sunday
Restraint 1 h Shaking 1 h	Hot air 10 min Cage Tilt	Shaking 1 h Odor 12 h	Restraint 1 h Hot air 10 min	Odor 12 h Cage Tilt	Water deprivation	Food deprivation
Shaking 1 h Odor 12 h	Hot air 10 min Restraint 1 h	Shaking 1 h Cage Tilt	Hot air 10 min Restraint 1 h	Odor 12 h Food deprivation	Cage Tilt	Restraint 1 h
Water deprivation Restraint 1 h	Shaking 1 h Cage Tilt	Hot air 10 min Odor 12 h	Restraint 1 h Shaking 1 h	Hot air 10 min water deprivation	Cage Tilt	Odor 12 h
Restraint 1 h Hot air 10 min	Shaking 1 h Odor 12 h	Restraint 1 h Cage Tilt	Hot air 10 min Shaking 1 h	Odor 12 h Cage Tilt	Water deprivation	Food deprivation
Shaking 1 h Cage Tilt	Hot air 10 min Water deprivation.	Restraint 1 h Odor 12 h	Hot air 10 min Shaking 1 h	Restraint 1 h Food deprivation	Odor 12 h	Cage Tilt
Shaking 1 h Hot air 10 min	Restraint 1 h Odor 12 h	Hot air 10 min Shaking 1 h	Restraint 1 h Food deprivation	Shaking 1 h water deprivation	Cage Tilt	Odor 12 h

### Weight and food consumption measurement

As part of the wellness supervision scale, mice were weighted two at 3 days a week. Food consumption was assessed in group housed mice, during the first 2 weeks of CRS protocol, every 2 days, by measuring the total consumption of each cage and dividing by the number of mice. This gives us and estimate of the average food consumption per mice.

### Open field test

This test was used to measure anxiety-like behavior, as mice are exposed to an open and novel environment and aversion to open spaces contrasts with the desire to explore the arena. Anxious mice will spend less time at the center of the arena than non-anxious mice. Mice were transferred into another room with uniform light and waited for at least 30 min before behavioral testing. Subsequently, they were placed in the corner of an open field box (45 × 45 cm) and allowed to explore freely for 10 min. The mice activity in the arena was recorded and then analyzed using an imaginary grid of 5 × 5 quadrants that allows for dissecting the arena into periphery and center. The apparatus was cleaned with 70% ethanol after each trial to eliminate olfactory cues. The number of entrances and the time spent at the center of the arena were analyzed by Kinoscope (version 3.0.4), an open-source computer software utilized in neuroscience (Kokras et al., 2017). Distance traveled and average velocity were obtained through analysis by ToxTrak software (Rodriguez et al., 2018). This test was applied 2 days after the end of the stress protocol.

### Forced swim test

This test was employed to measure despair, one of the signs of depressive behavior. It consisted of forcing animals to swim in a cylinder with ¾ of water from which they cannot escape. An indicator of the state of hopelessness faced with the impossibility of escape was visualized as an increase in the time of immobility of the rodent in the water. Mice were transferred into another room with uniform light and waited for at least 30 min before behavioral testing, and then they were placed in a transparent acrylic cylinder filled with water at 22°C. The test lasts 6 min, of which the first 2 min were not considered for the evaluation of the animal’s behavior but were used as a settling-in period. The behavioral parameters assessed included swimming or fighting time and immobility. The duration of swimming or immobility time was recorded manually using the Kinoscope software (version 3.0.4). This test was applied 2 days after the OFT.

### Treg isolation

Treg cells were isolated from the spleens of control C57BL/6 adult male mice, by magnetic separation using the Dynabeads™ Regulatory CD4^+^/CD25^+^ T Cell Kit (Invitrogen, ThermoFisher, Carlsbad, CA, United States), according to the manufacturer’s instructions. Briefly, spleens were collected in phosphate buffered saline (PBS) 1X supplemented with 5% of fetal bovine serum (FBS) (Gibco, Life Technologies, Paisley, United Kingdom) immediately after euthanasia and disaggregated by pressing a 1 mL syringe plunger against a 70 μm cell strainer. The suspension was incubated to ACK lysis buffer (Gibco, Life Technologies, Paisley, United Kingdom) for 5 min at ambient temperature for red blood cells lysis, diluted with PBS 1X, and centrifuged at 2000 rpm for 5 min. Supernatants were discarded and pellets were resuspended in Ca^++^ and Mg^++^ free PBS 1X, supplemented with 2 mM EDTA at a ratio of 1 mL per 10^6^ cells. Suspension was incubated with an antibody mixture provided in the kit, at 4°C for 20 min. Afterward, cells were washed with PBS 1X and incubated for 15 min at ambient temperature with pre-washed depletion Dynabeads, and then placed for 2 min in the DynaMag™-15 (Invitrogen, ThermoFisher, Carlsbad, CA, United States). Supernatant was collected and transferred into new tubes for incubation with FlowComp Mouse CD25 antibody, provided in the kit, at ambient temperature for 20 min. Cells were washed and incubated with pre-washed FlowComp Dynabeads for 15 min at ambient temperature. After, cells were placed for 2 min in the DynaMag™-15 (Invitrogen, ThermoFisher, Carlsbad, CA, United States), and supernatant containing effector T cells were eliminated. The pellet was washed and incubated with FlowComp release buffer for 20 min at ambient temperature and placed again for 2 min in the DynaMag™-15. Supernatant containing Treg cells were collected, centrifuged at 2000 rpm, and resuspended in PBS 1X. The complete procedure was done under a cell culture biosafety cabinet to preserve sterility.

### Treg adoptive transfer

After the third week of CRS protocol or the fourth week of CUS protocol, some mice were randomly selected to receive a single dose of 7^*^10^4^ Treg cells isolated from control adult mice by magnetic separation in 100 μL of sterile PBS 1X, or vehicle through an intravenous injection into the mice tail vein. Although usually, Treg cells dose in adoptive transfer experiments varies between 5^*^10^5^ and 1^*^10^6^ cells, this is equivalent to 4 or 8 times the amount of circulating Treg estimated by other (which is approximately 1.2^*^10^4^) (Churlaud et al., 2015; Bansal et al., 2019). Considering potential translation to the clinic, where values close to physiological ones are preferred, we decided to use approximately 50% of circulating Treg cells, like other studies (Landman et al., 2018; Yang et al., 2024).

Mice were immobilized by placing them into a mice restrainer holder (RWD, LifeScience, China), with the tail exposed to red light to facilitate tail vein dilation. Tails were disinfected with 70% ethanol and Treg cells injections were performed using a syringe with a 27G needle. On the day of the injection mice were not subjected to movement restriction to not increase the stress levels. The selected time points for each protocol respond to our interest in evaluating the therapeutic effect of Treg cells, considering a sufficient time for stress exposure prior to Treg administration and a sufficient time for potential Treg cells effects to take place and be detected. However different time points could have been explored.

### Tissue collection

Two to three days after the last behavioral test was performed, mice were euthanized by cervical dislocation after general anesthesia with a mixture of ketamine and xylazine. Immediately, 50 μL of blood was collected with a micropipette, from the thoracic cavity in Eppendorf tubes containing 20 μL of heparin solution (5,000 UI/mL, Laboratorio Sanderson, Chile). Later, 100 μL of ACK lysis buffer (Gibco, Life Technologies, Paisley, United Kingdom) was added to incubate at 37°C for 10 min. Washing steps were performed with PBS 1X supplemented with 5% of FBS (Gibco, Life Technologies, Paisley, United Kingdom). Spleen were also collected, by making an incision in the left lower side of each mouse and placed in Eppendorf tubes containing 500 μL of PBS 1X supplemented with 5% of FBS (Gibco, Life Technologies, Paisley, United Kingdom), on ice. Brains were carefully removed from the skulls and placed into a mouse brain slicer matrix for 1 mm coronal sections (RWD, LifeScience, China). A center slice was obtained from each brain, containing the hippocampal region, for fixation or protein isolation.

### Flow cytometry

Peripheral blood mononuclear cells (PBMC) and splenocytes were stained with anti-CD4-APC (1:100, Biolegend, San Diego, CA, United States), anti-CD25-PECy7 (1:200, BDBioscience) anti-CD44-PerCP (1:100, Biolegend), diluted in PBS 1X supplemented with 5% of FBS (Gibco, Life Technologies, Paisley, United Kingdom), by incubating in the dark at 4°C for 30 min. For intracellular staining with anti-FOXP3, permeabilization and fixation were performed by incubating with eBioscience FOXP3/transcription factor staining Buffer set (Invitrogen, ThermoFisher, Carlsbad, CA, United States), at 4°C in the dark for 45 min, according to the manufacturer instructions. Later, cells were incubated with anti-FOXP3-PE (1:100, Invitrogen, ThermoFisher, Carlsbad, CA, United States) at 4°C for 30 min in the dark. Cells were washed, resuspended with PBS 1X, and stored at 4°C until their analysis in a FACS Canto II flow cytometer (BD BioScience, San Diego, CA, United States). Subpopulation analysis was done using FlowJow software, version 10.6 (Tree Star).

### Brain histological processing and immunohistochemistry

Collected brain slices were immediately fixed in Bouin’s solution (acetic acid 5%, formaldehyde 9%, picric acid 0.9%) by immersion at 4°C for 72 h. Tissue was dehydrated in a battery of ascendent ethanol (Merck, Darmstadt, Germany), ending with xylol (Winkler, Chile), and included in histological paraffin (Invitrogen, ThermoFisher, Carlsbad, CA, United States) to obtain blocks. Paraffin blocks were cut into 4 μm sections and mounted on glass slides. Staining was performed with Hill Hematoxylin (Merck, Darmstadt, Germany) to identify the region of interest. In the selected tissue sections immunohistochemistry was performed for microglial cells, with anti-Iba-1 and proliferating cells with PCNA. Tissue sections were deparaffinized and endogenous peroxidase activity was quenched using 30% hydrogen peroxide (Winkler, Chile). Washes were performed with TrisPO_4_ buffer 1X (Tris 0.12 M, KH_2_PO_4_ 0.035 M, NaCl 1.19 M, Na_2_HPO_4_^*^2H_2_O 0.08 M) buffer 1X. Incubation with anti-Iba-1 (1:400, Wako, Fujifilm) and anti-PCNA (1:200, Invitrogen, ThermoFisher, Carlsbad, CA, United States) occurred overnight at room temperature under a humid chamber. Following primary antibody incubation, sections were washed with TrisPO_4_ buffer and incubated with mouse and rabbit HRP-conjugated secondary antibody for 2 h (1:200, Jackson Immunoresearch, West Grove, PA, United States). After the final wash, sections were incubated with ImmPACT^®^ DAB substrate Kit (Vector Laboratories, Newark, CA, United States) for 5 min in the dark, and counter-stained with Hill Hematoxylin.

### Image processing and morphometric analyses

Microglia number and morphology were analyzed in the dentate gyrus (DG) of the hippocampus, specifically the region below the hippocampal fissure, containing both the granule and the molecular layers. The hippocampus region was selected between Bregma −1.55 and − 2.03. Whole slide digital images were taken using NIKON Eclipse microscope (TI) at 100x magnification, while representative images of microglia in DG were captured with Olympus BX41 microscope at 1000x magnification. Skeleton analyses were performed to examine the morphology of Iba-1 positive cells, using FIJI (Schindelin et al., 2012) and ImageJ software. Briefly, images at 1000x magnification of Iba-1 labelled cells were imported into FIJI, and DAB marked zones were obtained after a color deconvolution step. For this analysis, five Iba-1 positive cells morphologically representative of the microglia observed in each section were randomly selected across DG sections for each mouse and analyzed blindly. Cell images were converted into binary images and skeletonized. First, using the AnalyzeSkeleton plugin, we performed a prune cycle using the shortest branch method to all skeletonized images, and then, the average branch length and branch number data given by the software was registered.

### RT-PCR analysis

Total RNA was extracted from the spleen using Trizol (Invitrogen, Thermo Fisher, Carlsbad, CA, United States) reagent, according to the manufacture instructions, and absorbance was measured at 260 and 280 nm in a microplate spectrophotometer UV–Vis EPOCH (Bio Tek Instruments Inc., Winooski, VT, United States) to quantify and determine quality. RNA was treated with DNase I (Sigma-Aldrich, St. Louis, MO, United States) to eliminate contaminant genomic DNA. Complementary DNA (cDNA) was prepared using an iScript cDNA synthesis kit (Bio-Rad, Hercules, CA, United States) from 2 μg total RNA. PCR amplifications were performed on a Real-Time PCR Detection System (QuantStudio 3 system, Applied Biosystems by Thermo Fisher Scientific) using the SYBR green SsoAdvanced universal super mix (Bio-Rad, Hercules, CA, United States) under the following conditions: initial activation at 95°C, followed by 40 cycles of amplification at 55°C for 30 s and 72°C for 30 s. Primers used were the following: TGF-β: forward 5´-TTTTGCTCCTGCATCTGGT-3′, reverse 5’-CCTGGTACTGTTGTAGATGGAA-3′; 18S: forward 5’-GCCCGAAGCGTTTACTTTGA-3′ and reverse 5’-TTGCGCCGGTCCAAGAATTT-3′. The fold change was calculated using the 2-ddCt method, normalized against the internal control, 18 s.

### Statistical analysis

Number of mice used in this study was calculated with a power of 0.8. Data was expressed as the mean ± SD. Both parametric and non-parametric tests were employed because the sample size was less than 30, despite exhibiting a normal distribution. To assess differences between two groups, independent samples *t*-tests and Mann–Whitney tests were utilized. For analyses involving more than two groups, one way ANOVA followed by a *post hoc* Tukey multiple comparison and Kruskal–Wallis tests were applied. Cohen’s d was used to calculate effect size, as a measure of the magnitude of the differences between two groups. This indicator was incorporated for its relevance to better understand and interpret pre-clinical and clinical data (Sullivan and Feinn, 2012). A Cohen’s d ≥ 0.8 indicates a large effect. All statistical analyses were made using GraphPad Prism 8.4 (Graph Pad Software Inc., La Jolla, CA, United States) and R software for Windows, version 4.3.3.

## Results

### Mice submitted to CRS protocol exhibit anxiety and depressive-like behavior and have lower frequencies of circulating Treg cells

**Figure 1:**
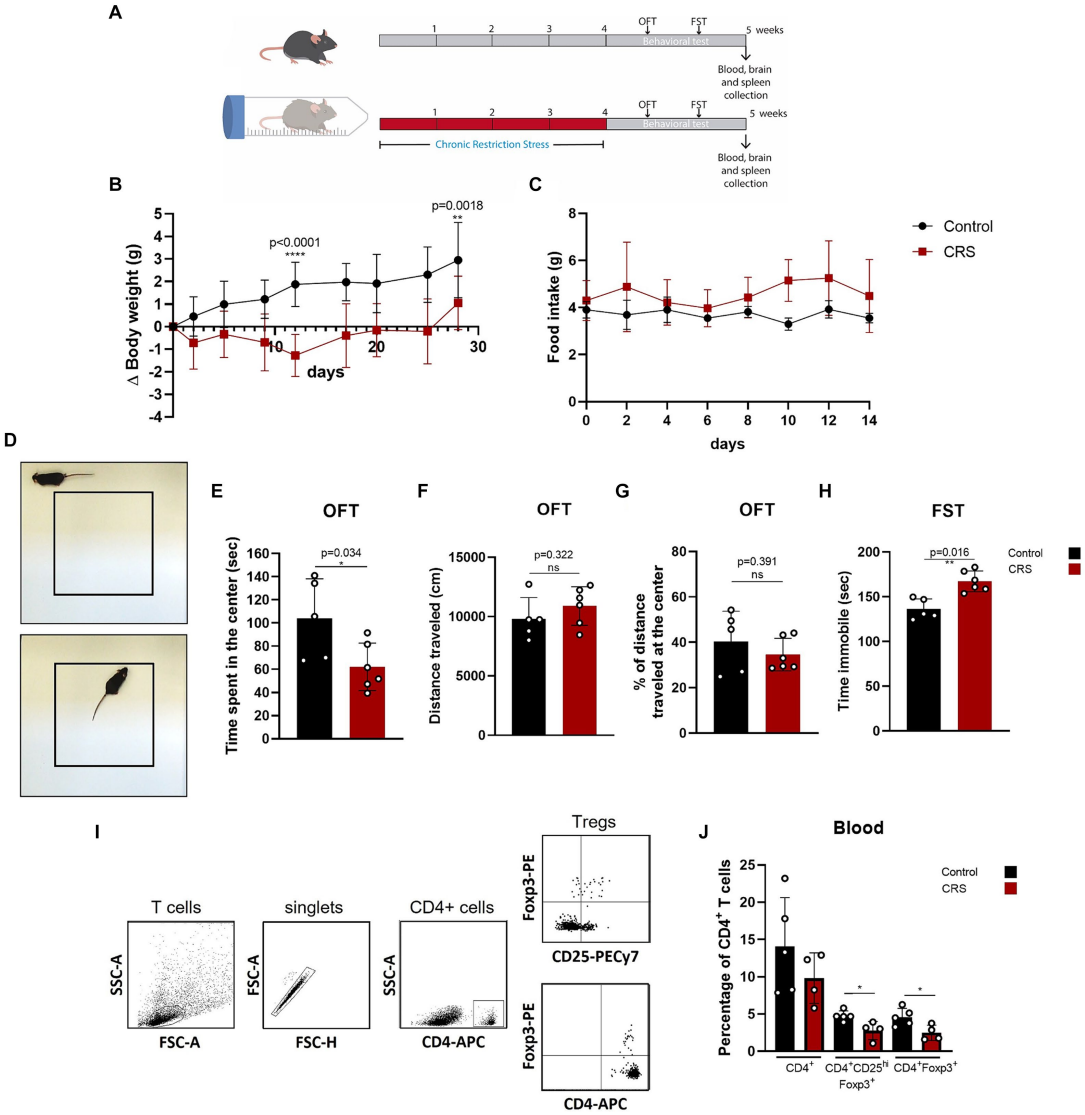
Mice submitted to CRS protocol exhibit anxiety and depressive-like behavior and have lower frequencies of circulating Treg cells. **(A)** Diagram depicting CRS protocol and behavioral testing. **(B)** Delta body weight curves for control (black) and CRS (red) mice. **(C)** Food consumption curves in control (black) and CRS (red) mice. **(D)** Representative images of the periphery and the center in the open field test (OFT). **(E–G)** Bar Graphs for time spent at the center of the arena, total distance traveled, and percentage of distance traveled at the center on the OFT in control (black) and CRS (red) mice. **(H)** Bar graph for time immobile in the FST in control (black) and CRS (red) mice. **(I)** Representative flow cytometry dot plots showing the gating strategy for defining CD4+ T cells and regulatory T cell populations. **(J)** Bar graph for the CD4+ T cells population frequencies in blood for control (black) and CRS (red) mice. The results are shown as the mean ± SD, from two independent experiments, with an *n* = 5 for control group and *n* = 6 for CRS. Student *t*-test was used to calculate statistical differences between group means. ^*^p < 0.05, ^**^p < 0.01, ^***^p < 0.001, ^****^p < 0.0001.

One of the simplest stress models for inducing anxiety and depression is CRS, which consists of immobilizing mice for a period of hours a day, repeatedly. This model has been used to study the effects of stress in the organism and to evaluate the efficacy of antidepressive drugs (Ampuero et al., 2015; Misztak et al., 2021). So far, one study has reported a decrease in Treg cells frequency in this model, demonstrating that Treg cells depletion promotes anxiety and depressive like-behavior (Kim et al., 2012). We induced chronic stress in adult C57BL/6 mice by CRS paradigm for 28 days and afterwards, we evaluated behavior and Treg cells in blood by flow cytometry (Figure 1A), which were statistically analyzed by parametric T Student Test and non-parametric Mann–Whitney U-Test. Mice submitted to CRS protocol show lower weight gain curves throughout the experimentation period (Figure 1B), as has been reported before (Wu et al., 2023), which are not due decreased food consumption (Figure 1C). Anxiety-like behavior was measured by the open field test (OFT) (Figure 1D), while depressive-like behavior was assessed by the forced swim test (FST). Stressed mice spent significant less time in the center of the OFT arena (control 103.8 ± 34.4 s vs. 62.3 ± 20.5 s; Cohen’s d size effect of 1.5), which is a sign of anxiety-like behavior as the fear for open and novel spaces is stronger that the desire to explore the arena (Figure 1E), with no differences in locomotion activity, as they traveled similar total distance (Figure 1F). We did not detect differences in the distance traveled at the center of the arena, as mice that spent more time in the center mostly performed grooming or explore this region moving slowly (Figure 1G). Stressed mice also had increased immobility time in the FST (control 136.2 ± 11.4 s vs. 167.3 ± 12.1 s; Cohen’s d size effect of 2.7), which is a sign of despair, indicative of depressive-like behavior (Figure 1H).

One week after CRS protocol ended, mice were euthanized and blood was collected for staining with fluorophore conjugated antibodies against CD4^+^, CD25^+^, and FOXP3 to see changes in circulating CD4^+^ Treg cells by flow cytometry (Figure 1I). Although by consensus it has been established that the identity of the Treg cells is defined by the expression of CD4, Foxp3 and CD25^hi^. CD25^+^ expression is actually heterogenous, as it depends on co-stimulator signaling by CD28 and IL-2, but importantly, it can be downregulated in Treg cells, without compromising their suppressive capacity (Tang et al., 2003; Nishioka et al., 2006; Tang et al., 2008), so this is why we decided to analyze Treg cells with and without CD25^+^ expression. Our analysis show that mice submitted to CRS have significantly lower frequencies of circulating Treg cells, compared to control mice (control 4.6 ± 1.2% vs. CRS 2.5 ± 1.0% of CD4^+^ T cells; Cohen’s d size effect of 1.6) (Figure 1J).

### Adoptive transfer of Treg cells reduces peripheral inflammation and reduces anxiety-like behavior in CRS mice

Because anxiety and depression have been linked to low grade inflammation, we hypothesized that compensating for the reduced frequency of circulating Treg cells could have an effect on inflammation signs and reduce anxiety and depressive-like behavior on CRS mice. Treg cells adoptive transfer has been tested before to alleviate severe immune responses, such as the ones observed in allogeneic transplantation (Trenado et al., 2006) or autoimmune disease (Lapierre et al., 2013; Beers et al., 2017). However, this strategy has not been used before for chronic low-grade inflammation underlying depressive and anxious disorders.

**Figure 2:**
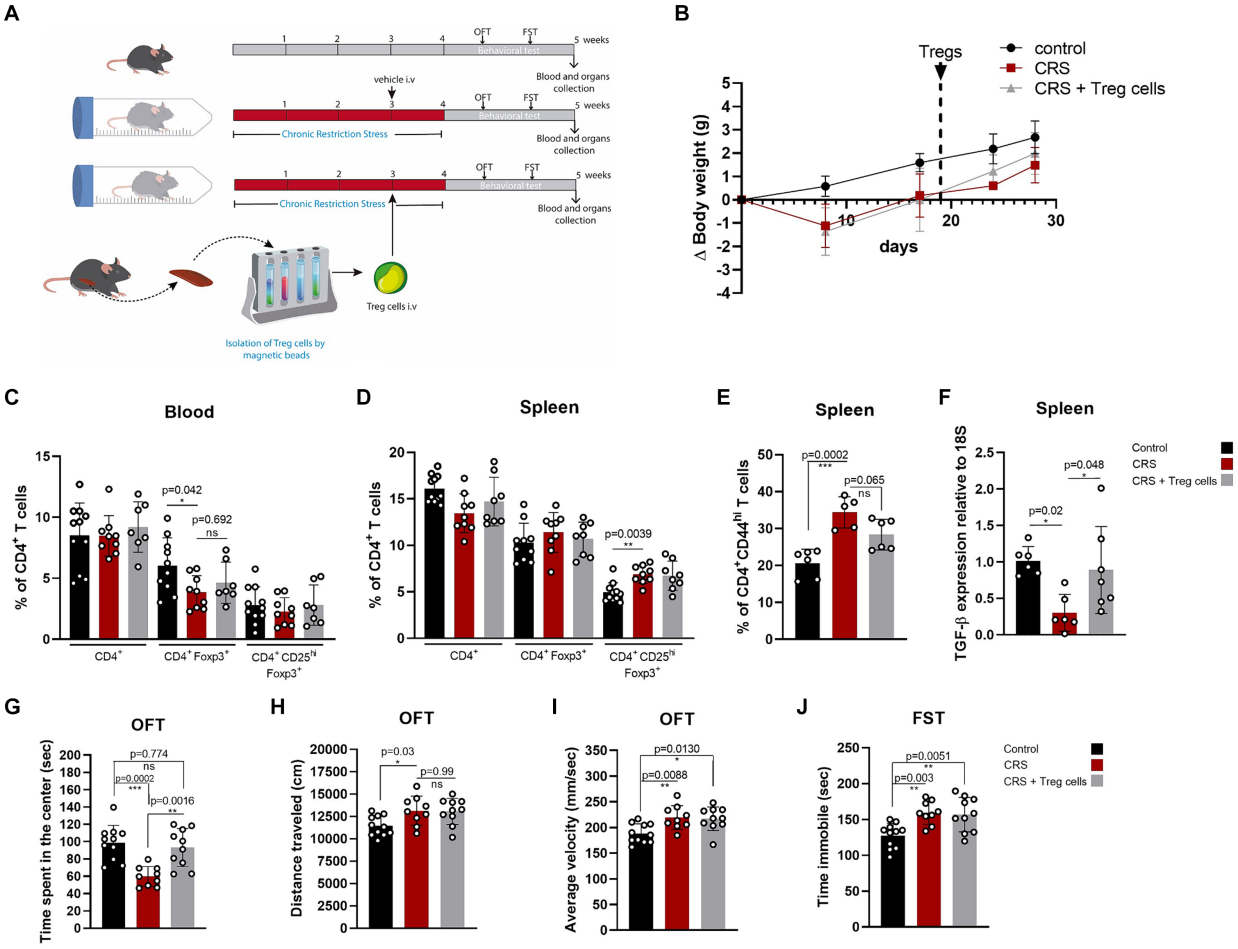
Treg cells adoptive transfer reduces peripheral inflammation and anxiety-like behavior in CRS. **(A)** Diagram depicting CRS protocol, Treg cells adoptive transfer, and behavioral testing. **(B)** Delta body weight curves for control (black), CRS (red), and CRS mice receiving Treg cells (gray). **(C–E)** Bar graphs for the CD4+ T cells population frequencies in blood **(C)** and spleen **(D,E)** for control (black), CRS (red), and CRS mice receiving Treg cells (gray). **(F)** Bar graph for TGF-β expression relative to 18S in the spleen of control (black), CRS (red), and CRS mice receiving Treg cells (gray). **(G–I)** Bar graphs for the variables time spent at the center of the arena **(G)**, distance traveled **(H)**, and average velocity **(I)** in the OFT, for control (black), CRS (red), and CRS mice receiving Treg cells (gray). **(J)** Time immobile in the FST in control (black), CRS (red), and CRS mice receiving Treg cells (gray). The results are shown as the mean ± SD, from three independent experiments, with an *n* = 11 for control group, *n* = 9 for CRS group and *n* = 10 for CRS-Treg cells group. One-way ANOVA was used to calculate statistical differences between group means. ^*^p < 0.05, ^**^p < 0.01, ^***^p < 0.001, ^****^p < 0.0001.

We injected CRS-stressed mice with a single dose of about 7 × 10^4^ freshly isolated Treg cells, or PBS at the end of the third week of CRS protocol (Figure 2A). Treg cells were isolated from control adult mice and infused through an intravenous injection into the mice tail vein. Body weight was controlled to detect detrimental effects of Treg cells administration, and no differences were observed between mice receiving the vehicle and Treg cells (Figure 2B). After CRS protocol and behavioral testing, mice were euthanized and blood, spleen, and brain were collected to study changes in T cell populations and neuroinflammation signs, which were statistically analyzed by ANOVA. Our results showed that Treg cells administration did not change the frequencies of circulating Treg cells (Figure 2C), nor Treg cells from the spleen (Figure 2D), after 2 weeks. However, the frequency of effector CD4^+^ T cells in the spleen, which are higher in mice subjected to CRS was decreased when mice received adoptive transfer of Treg cells (control 20.7 ± 3.7 vs. CRS 34.4 ± 4.2 vs. CRS + Treg cells 28.3 ± 4.2% of CD4 + CD44^hi^; Cohen’s d size effect of 3.5 between control and CRS and 1.44 between CRS and CRS + Treg cells) (Figure 2E). Moreover, we detected a reduction in the expression of the anti-inflammatory cytokine TGF-β by RT-PCR in the spleen that was increased with Treg administration (Cohen’s d size effect of 3.1 between control and CRS and 1.2 between CRS and CRS + Treg cells) (Figure 2F). These results suggest that Treg cells transfer to stressed mice decreases peripheral inflammation.

Anxiety and depressive-like behavior analyzed by OFT and FST shows that Treg cells administration increased the time spent in the center of CRS mice, resembling the values of the control group (control 101.7 ± 18.6 s vs. CRS 59.8 ± 11.5 s vs. CRS + Treg cells 93.3 ± 22 s; Cohen’s d size effect of 2.7 between control and CRS and 1.9 between CRS and CRS + Treg cells) (Figure 2G). We did not observe differences in the distance traveled and average velocity (Figures 2H,I). Additionally, no changes were observed in the immobility time on the FST after Treg cells administration (Figure 2J).

### Adoptive transfer of Treg cells does not reduce anxiety nor depressive-like behavior in CUS

**Figure 3:**
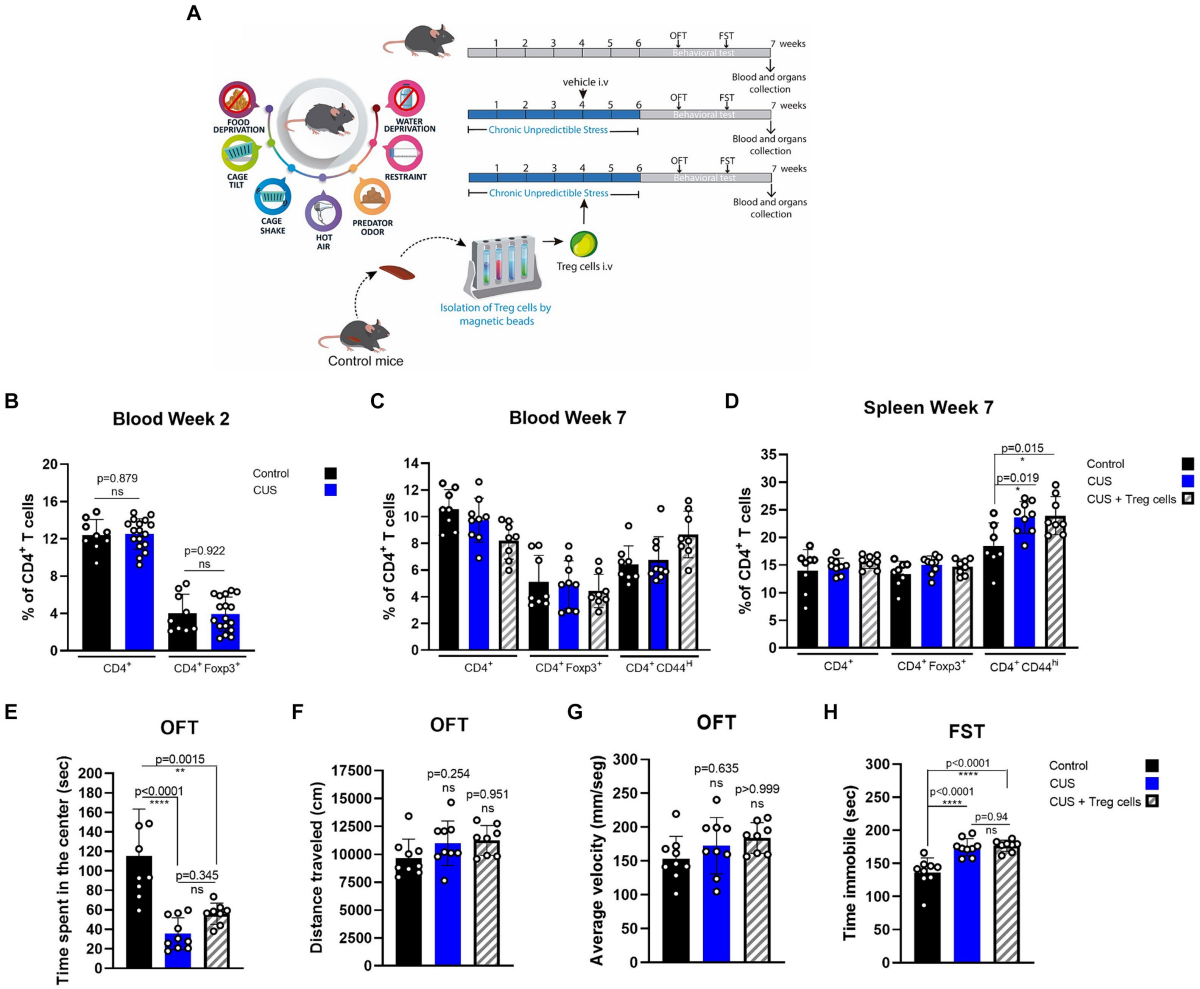
Treg cells adoptive transfer does not reduces anxiety-like behavior in CUS. **(A)** Diagram depicting CUS protocol, Treg cells adoptive transfer, and behavioral testing. **(B–D)** Bar graphs for the CD4+ T cells population frequencies in blood **(C,D)** and spleen **(E)** for control (black), CUS (blue), and CUS mice receiving Treg cells (dashed). **(E–G)** Bar graphs for the variables time spent at the center of the arena **(E)**, distance traveled **(F)**, and average velocity **(G)** in the OFT, for control (black), CUS (blue), and CUS mice receiving Treg cells (dashed). **(H)** Time immobile in the FST in control (black), CUS (blue), and CUS mice receiving Treg cells (dashed). For flow cytometry and behavioral analysis, the results are shown as the mean ± SD, from two independent experiments, with an *n* = 9 for control and CUS groups and *n* = 8 for CUS-Treg cells group. One-way ANOVA was used to calculate statistical differences between group means. ^*^p < 0.05, ^**^p < 0.01, ^***^p < 0.001, ^****^p < 0.0001.

We tested if peripheral Treg cells administration could also influence inflammatory signs and anxiety or depressive-like behavior in another widely used model of chronic stress, known as chronic unpredictable stress (CUS), where mice are submitted to a series of uncomfortable stimuli daily, distributed randomly (Table 1; Figure 3A). Same as before, we injected CUS-stressed mice with a single dose of about 7 * 10^4^ freshly isolated Treg cells, or PBS at the end of the fourth week of CUS protocol (Figure 3A). Because CUS paradigm has a longer duration, compared to CRS, and Treg cells frequency might be dynamic over time, we included blood analysis at two different times (2 and 7 weeks after CUS initiated). All variables were statistically analyzed by ANOVA. In this model, we did not detect differences in CD4^+^ T cells and Treg cells in blood between control and stressed mice, after 2 weeks of stress nor at the end of the stress protocol (Figures 3B,C). However, we did observe an increase in the frequency of effector CD4^+^ T cells in the spleen after CUS (Figure 3D) which was not diminished in mice that received Treg cells (control 18.4 ± 4.2% vs. CUS 23.6 ± 2.9% vs. CUS + Treg cells 23.9 ± 3.4% of CD4^+^CD44^hi^; Cohen’s d size effect of 1.5 between control and CUS and 1.45 between CUS and CUS + Treg cells).

CUS mice spent less time at the center of the arena in the OFT, and although an increasing trend was observed for CUS mice that received Treg cells it was not statistically significant by ANOVA test, (control 115.0 ± 48.5 s vs. CUS 35.6 ± 16.3 s vs. CUS + Treg cells 56.0 ± 10.9 s; Cohen’s d size effect of 2.2 between control and CUS and 7.5 between CUS and CUS + Treg cells) (Figure 3E). We did not observe differences in the other OFT parameters distance traveled and average velocity (Figures 3F,G) nor in the immobility time (Figure 3H) when Treg cells were administered.

### Treg cells adoptive transfer modulates microglia number in the hippocampus dentate gyrus of CRS and CUS mice, restoring their arborization only in CRS model

**Figure 4:**
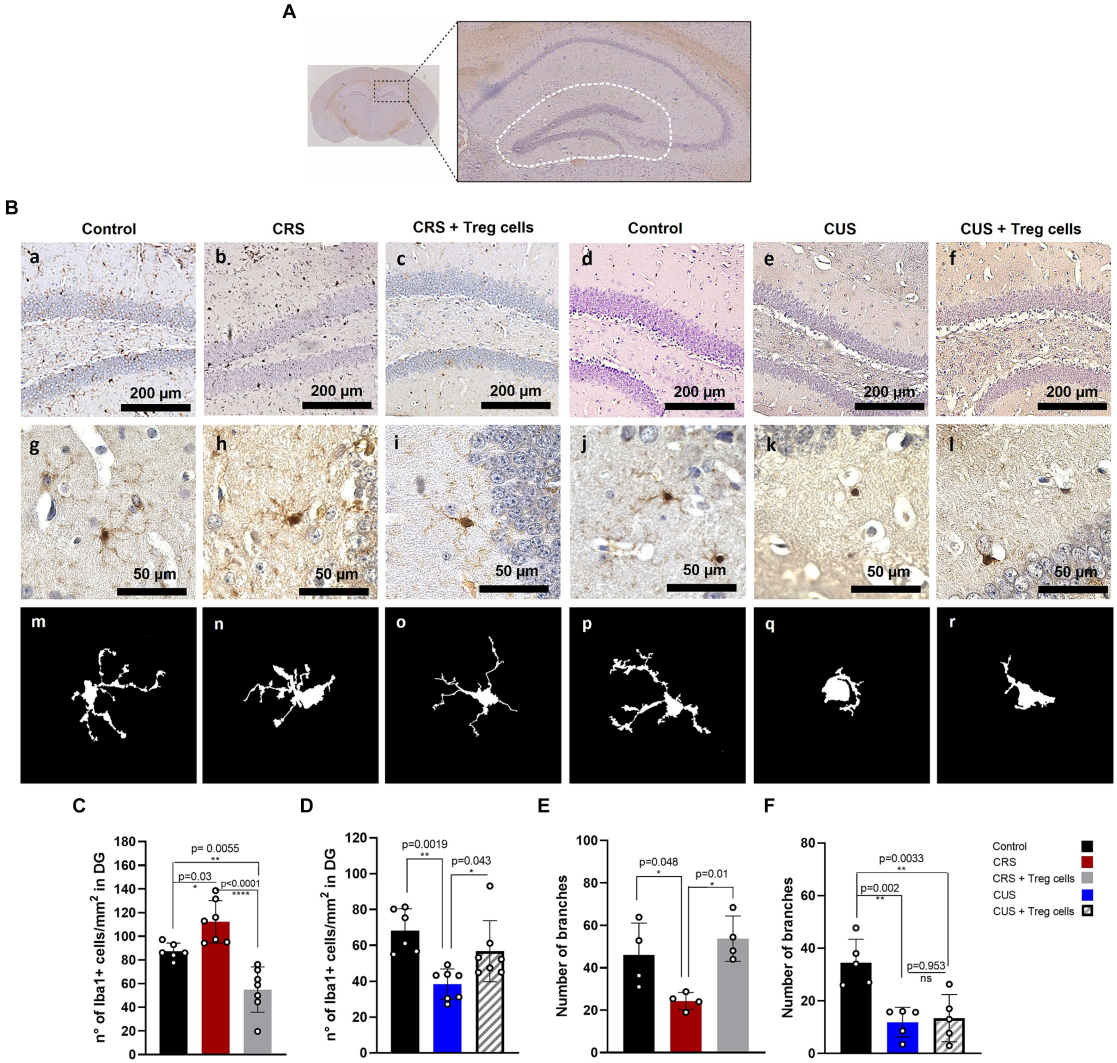
Treg cells adoptive transfer modulates microglia number in the hippocampus dentate gyrus of CRS and CUS mice, restoring their arborization only in CRS model. **(A)** Representative image of the hippocampal area analyzed. **(B)** Representative immunohistochemistry for Iba-1 in brain sections containing the hippocampal DG region of control group for CRS protocol **(A)**, CRS **(B)**, CRS receiving Treg cells **(C)**, control group for CUS protocol **(D)**, CUS **(E)** and CUS mice receiving Treg cells **(F)**, with higher magnification in g-l and representative images of individual microglia deconvoluted and skeletonized with ImageJ (m–r). **(C–F)** Bar graphs for quantification of microglia number in the DG region **(C,D)** and number of branches **(E,F)** in control (black), CRS (red), CRS receiving Treg cells (grey), CUS (blue) and CUS mice receiving Treg cells (dashed). Images were taken with a 100X magnification in a–e or 400X magnification in (f–j). Scale bars: 200 μm (a–f) and 50 μm (g–l). The results are shown as the mean ± SD from two independent experiments, with an *n* = 7 for control in CRS protocol, *n* = 6 for CRS, *n* = 7 for CRS mice receiving Treg cells, *n* = 6 for control in CUS protocol, *n* = 7 for CUS and *n* = 7 for CUS mice receiving Treg cells. For microglia morphological analysis, results are shown as the mean ± SD of 5 randomly selected microglia from five different mice for each group. One-way ANOVA was used to calculate statistical differences between group means. ^*^p < 0.05, ^**^p < 0.01, ^***^p < 0.001, ^****^p < 0.0001.

To explain the improvement in the anxiety parameter of time spent in the center of the arena, in both CRS and CUS mice that received Treg cells adoptive transfer, we analyzed changes in microglia number and morphology, in the DG of the hippocampus brain region (Figure 4A), as an indicator of neuroinflammation (Wang et al., 2018; Du Preez et al., 2020; Machado-Santos et al., 2021). Statistical differences were evaluated by ANOVA. Our immunohistochemistry results show that CRS protocol increases the number of Iba-1 positive cells/mm^2^ in the DG, while Treg cells peripheral administration into CRS mice significantly reduces it, observing lower values than in the controls (control 87.3 ± 6.9 vs. CRS 112.2 ± 17.9 vs. CRS + Treg cells 54.9 ± 19.1 n° of Iba-1^+^ cells/mm^2^; Cohen’s d size effect of 1.1 between control and CRS, 1.9 between CRS and CRS + Treg cells, and 1.33 between control and CRS + Treg cells) (Figures 4Ba–c,C). In mice submitted to CUS protocol, on the other hand, we observed a significant decrease in microglia number in the DG, while Treg administration into CUS mice increased their number, although not reaching control levels (control 68.3 ± 12.1 vs. CUS 38.4 ± 8.5 vs. CUS + Treg cells 56.7 ± 17 of Iba-1^+^ cells/mm^2^; Cohen’s d size effect of 2.7 between control and CUS, 1.3 between CUS and CUS + Treg cells, and 0.9 between control and CUS + Treg cells) (Figures 4Bd–f,D).

In addition, CRS protocol also reduced microglia arborization, by decreasing the number of branches, and this was restored by Treg cells administration (control 46.0 ± 15.1 vs. CRS 24.3 ± 4 vs. CRS + Treg cells 53.7 ± 10.7; Cohen’s d size effect of 1.5 between control and CRS and 1.9 between CRS and CRS + Treg cells) (Figures 4Bg–i,E). CUS protocol, on the other hand, drastically reduced microglia arborization, observing an ameboid phenotype that could not be rescued by Treg cells administration (Figures 4Bj–l,F).

**Figure 5:**
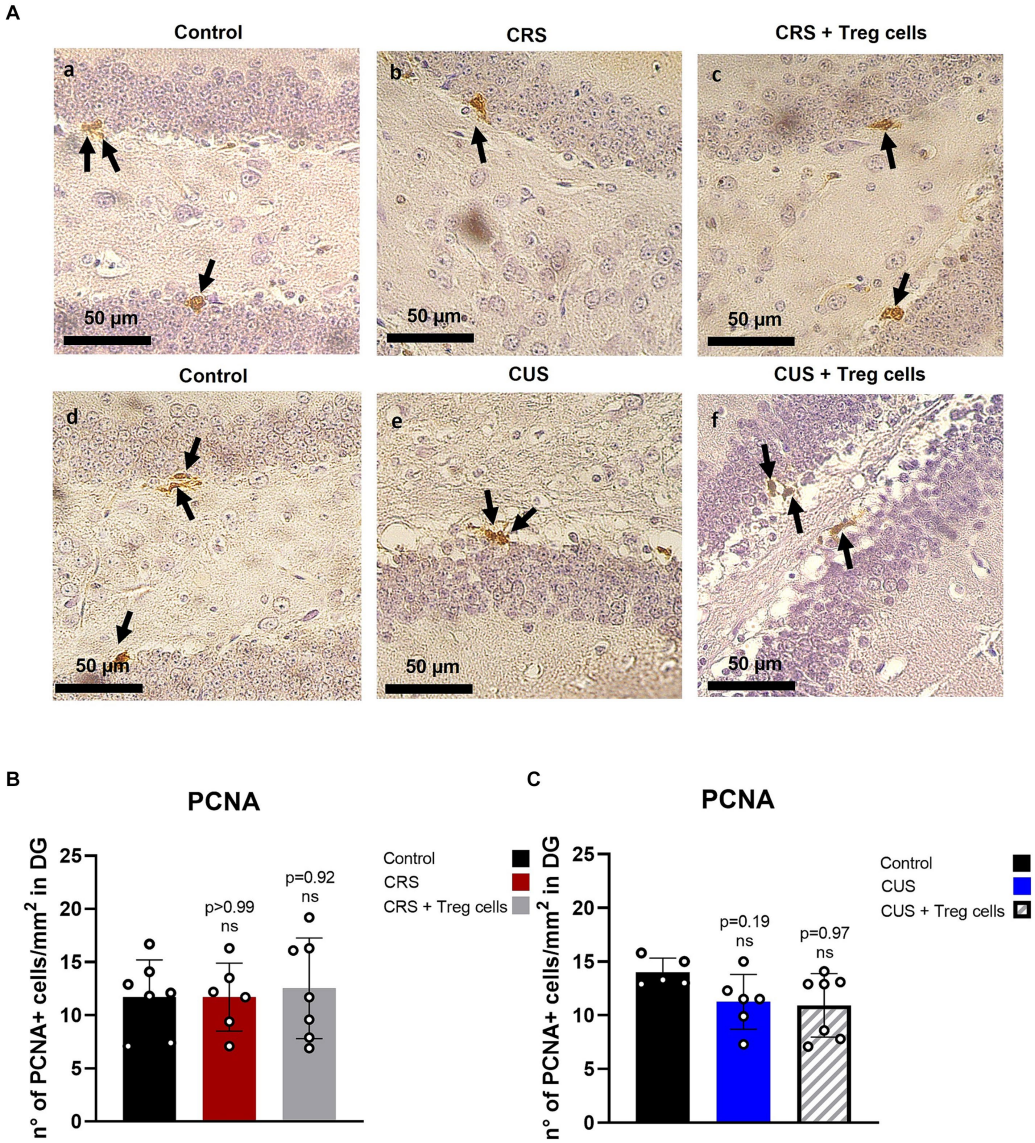
Treg cells adoptive transfer does not affect proliferation in the hippocampus dentate gyrus. **(A)** Representative immunohistochemistry for PCNA in brain sections containing the hippocampal DG region of control group for CRS protocol (a), CRS (b), CRS receiving Treg cells (c), control group for CUS protocol (d), CUS (e) and CUS receiving Treg cells (f). Arrows point out PCNA+ cells. **(B,C)** Bar graph for quantification of PCNA+ cells in the DG region for CRS **(B)** and CUS protocol **(C)** in control (black), CRS (red), CRS receiving Treg cells (grey), CUS (blue) and CUS mice receiving Treg cells (dashed).The results are shown as the mean ± SD from two independent experiments, with an *n* = 7 for control in CRS protocol, *n* = 6 for CRS, *n* = 7 for CRS mice receiving Treg cells, *n* = 5 for control in CUS protocol, *n* = 6 for CUS and *n* = 7 for CUS mice receiving Treg cells. Scale bars: 50 μm. One-way ANOVA was used to calculate statistical differences between group means. ^*^p < 0.05, ^**^p < 0.01, ^***^p < 0.001, ^****^p < 0.0001.

It has been previously shown that stress reduces hippocampal neurogenesis (Chesnokova et al., 2016), and this has been linked to depressive and anxiety-like behavior, so we analyzed if Treg cells administration could somehow influence proliferation in this neurogenic niche, by performing immunohistochemistry for the proliferation marker PCNA (Figure 5A). Unfortunately, despite what has been shown in the literature, we could not detect a significant decrease in the number of PCNA+ cells in the DG after CRS or CUS protocols. Treg cells peripheral administration, on the other hand, did not show a significant change (Figures 5A–C).

## Discussion

Several studies in patients with MDD not under antidepressive treatment, have shown a decrease in circulating Treg cells (Li et al., 2010; Chen et al., 2011; Grosse et al., 2016b). On the other hand, patients under antidepressive therapy show an increased frequency of Treg cells in circulation (Himmerich et al., 2010; Mohd Ashari et al., 2019; Alvarez-Mon et al., 2020). In animal models of depression, Treg cells frequency has been previously evaluated in CRS model, finding a reduction in circulation (Kim et al., 2012), and in CUS model, observing an increase in the spleen, at early time points (Hong et al., 2013). In this study, we demonstrated that adult male mice subjected to CRS exhibited lower frequencies of circulating Treg cells, resembling what was observed in many depressed patients and the previous report by Kim et al. (2012). Our results, also indicate that CRS-stressed mice showed increased Treg cells frequencies in the spleen, correlating with the observations made by Hong et al. (2013) in CUS model.

Considering the evidence pointing out that depressive-like behavior might partially be explained in terms of low-grade systemic and neuroinflammation status, strategies for restoring immunological balance have been proposed in the context of mood disorders, such as Treg cells expansion by low-dose IL-2 (Ellul et al., 2018). However, no study has yet evaluated the effect of the direct administration of Treg cells on anxiety and depression-like behavior, in stressed mouse models.

In this study we decided to test the effect of a single IV administration of Treg cells in mice submitted to CRS and CUS. Despite both models are widely used as chronic stress paradigms to induce anxiety and depressive-like behavior, the effect they have on mouse physiology could not necessarily be the same. This can be explained by differences in the severity and duration of each paradigm. CRS uses a single stressor (movement restriction) daily, for a shorter period, while CUS uses multiple stressors randomly for a longer period, making more difficult for the animals to habituate to the stress. Differences in behavioral response, cortisol secretion and serotonin levels in the brain have been found between social isolation and CUS, for instance (Lee et al., 2020). In this work we found differences in behavior, circulating Treg frequencies and microglia number and morphology between CRS and CUS models. Importantly, we also observed differences in these parameters after Treg cells administration. Based on our bibliographic search, this work is one of the first to highlight the differences at the level of microglial activation between the two models.

In the CRS protocol, mice receiving Treg cells showed a decrease in the frequency of effector CD4^+^ CD44^hi^ T cells and a recovery of TGF-β expression in the spleen, which could be interpreted as anti-inflammatory response (Pure and Cuff, 2001). Treg cells administration also reduced anxiety-like behavior measured by the OFT, with no effect on depressive-like behavior measured by the FST.

We evaluated changes in microglia after CRS protocol, observing that adult male mice exposed to CRS increases the number of microglia in the hippocampus, also exhibiting a less arborized phenotype, indicative of microglia reactive to stress. Administration of peripheral Treg cells decreased microglia number beyond control levels, and restored microglia arborization, like what was observed in controls. However, because there was no follow-up of the animals beyond 2 weeks post-treatment, we do not know if this reduction in microglia returns at any point to the normal levels observed in control mice. Based on the different analysis performed, the anxiolytic effect of Treg cells administration in the CRS protocol could be explained by the increase in TGF-β and the reduction in microglia. TGF-β release by Treg cells is one of its classical mechanisms used to suppress innate and adaptive immune cells (Li et al., 2007). Studies in humans have shown that TGF-β levels in plasma are decreased in patients with depression (Lin et al., 2024), while studies on mice models of stress have also detected decreased levels of TGF-β in the hippocampus (Baghaei Naeini et al., 2023) and basolateral amygdala (Zhong et al., 2022). Moreover, TGF-β overexpression in the amygdala of mice that develops anxiety-like behavior after being exposed to LPS during neonatal stages, normalized their behavior (Zhong et al., 2022). Curcumin treatment of mice exposed to CUS protocol reduces anxiety and depressive-like behavior also increasing the expression of TGF- β in serum and prefrontal cortex (Yang et al., 2023). On the other hand, TGF- β has been shown to have a pivotal role for microglia homeostasis, preventing its exaggerated activation under physiological conditions (Spittau et al., 2020), which allows us to connect both results found in the CRS model after the administration of Treg cells.

In the CUS protocol, which may be considered a more stressful model, mice spent less time in the center in the OFT and more time immobile in the FST, than CRS mice. A single Treg cells administration, at the dose evaluated did not have an effect on anxiety nor depressive-like behavior. When we looked at microglia number and arborization, we observed that CUS mice, contrary to the CRS model had a considerably lower number of microglia in the hippocampus, and their phenotype was more ameboid, suggesting a more reactive status to stress. Based on the results from both models it appears that anxiety-like behavior does not directly correlate with microglia number in the hippocampus, but it does correlate with the reduction in arborization, being more intense in the CUS model. On the other hand, Treg administration only affected microglia number, but not the arborization which could be related to the absence of anxiolytic effect observed in the CUS protocol. Microglia number and morphology alterations have been previously described in neurodegenerative and neuropsychiatry disorders, being considered as a relevant hallmark of central nervous system (CNS) pathogenesis (Mondelli et al., 2017; Hickman et al., 2018; Maras et al., 2022; Gao et al., 2023). In the context of stress-induced depression and anxiety models, some studies have shown increased microglia number (Tynan et al., 2010) and higher area (Wohleb et al., 2012; Du Preez et al., 2021) while others show reduced microglia number and reduced arborization (Tong et al., 2017). This inconsistency might relate to the intensity and chronicity of the stressor used in animal models, as well as the brain area evaluated, the rodent type, strain, age, and gender. However, functional analysis of these stress-responding microglia is needed to better understand when the initial adaptive response becomes detrimental to the CNS.

The differences observed in the effectiveness of Treg cell administration on the recovery of microglia arborization, between the two models, may be due to the differences in the intensity of the stress levels to which the CRS and CUS models are subjected. In a context of greater intensity of stress, as described for the CUS model, it is possible to suggest that the administration of a single IV dose of Treg cells is not sufficient, and therefore, it is necessary to increase the dose and/or the frequency of the administration of Treg cells.

In this study, the capacity of administrated Treg cells to cross the blood brain barrier (BBB) and infiltrate the hippocampus, was not evaluated, so we cannot determine if the effects observed in microglia were direct or not. However, a study in mice has shown that following experimental stroke, Treg cells infiltrate into the brain, which can promote a reparative phenotype in microglia, through the secretion of osteopontin (Shi et al., 2021). Although some studies have shown that BBB is compromised in chronic stress models, allowing the infiltration of Th17 cells in the hippocampus (Peng et al., 2022), whether Treg cells can also enter the brain or if brain resident Treg cells are somehow activated remains to be determined. On the other hand, diverse evidence suggests that chronic stress modifies the structure of the intestinal epithelial barrier, suppresses the production of tight junction proteins, and increases intestinal permeability (Li et al., 2023). Bacterial translocation across the intestinal barrier may trigger inflammatory reactions in the circulatory system that compromise the BBB (Kelly et al., 2015). Moreover, bacterial translocation through the intestinal barrier has the ability to stimulate CNS microglia and release cytokines that promote inflammation (Alexandrov et al., 2020; Liu et al., 2024). Despite that we did not evaluate intestinal barrier permeability or gut microbiota, we cannot rule out this is also happening in our models. It has been described that Treg cells support the integrity of the barrier (Juanola et al., 2018). For this reason, it is a possibility that Treg cell administration effect over anxiety-like behavior could be due by an indirect effect over gut permeability.

This work highlights the fact that different stress models can induce similar behavior, while exhibiting important differences at the brain level, such as microglia reactivity to stress. This could explain why pharmacological treatment might have different efficacy in human patients.

Our results continue to corroborate the long-standing evidence favoring the contribution of immune system alterations in the pathophysiology on depression and anxiety, and moreover posits Treg-based therapy as an alternative worthy of continued study in the future. Noteworthy, other works on Treg biology for neurodegenerative or autoimmune diseases affecting the brain have used Treg cells depletion approaches (Dansokho et al., 2016; Yang et al., 2023) or direct Treg cells administration into the brain (Park et al., 2023), but in this study, we have shown that peripheral administration of Treg cells can have anti-inflammatory effects as well, bringing the gap closer to the clinical translation. Although our results on Treg cells effect over anxiety-like behavior are preliminary, we hope this promotes more pre-clinical studies that can deepen into the cellular and molecular mechanism behind it, with the possibility to move forward into clinical trials.

One confounding variable that was not measured in this study was the basal level of stress in the animals used. Although all the mice were housed under the same environmental conditions and veterinarian care, individual differences on cortisol levels and behavior could have existed before stress induction. The analysis of these parameters before stress protocols could have provided valuable information for mice inclusion and randomization into the different groups. In addition, despite we did not find a reduction in food consumption that could explain lower body weight in CRS mice, because we did not measure food intake continuously during the light and dark phases of the day, we cannot rule out if CRS mice start to eat sooner that control mice, in response to immobilization -induced anxiety.

One of the limitations of our experimental design in the absence of a control group, without stress, receiving Treg cells administration. The absence of this group does not allow us to know if Treg cells can have an anxiolytic effect on controls and therefore do not allow us to be certain if the effect we are seen on stress mice is being sub estimated or over estimated. This group was not originally conceived as part of the experimental design, because of the safety profile previously described in the literature by Treg cells in different pathophysiological scenarios, where this control group is not included (D'Alessio et al., 2009; Van Herck et al., 2020; Amini et al., 2022; Yang et al., 2022; Bluestone et al., 2023). However, we acknowledge that incorporation of this control group in future studies will be fundamental to determining dose response curves.

We recognized that a technical limitation of our study is that a wider battery of behavioral tests could be employed to analyze anxiety and depressive-like behavior, such as elevated plus maze and sucrose preference test, which would have strengthened our conclusions. In addition, concerns about FST have been raised in the last years in terms of its validity and severity (Molendijk and de Kloet, 2022), but this is still a matter of debate (Sewell et al., 2021; Becker et al., 2023). Other limitation of our study is the inconsistency in immune cell markers usage across experiments, such as the lack of CD44 marker in the blood samples of the CRS model and the lack of CD25 marker in the blood samples of the CUS model. In the case of CD25, we faced logistical difficulties as the antibody we were using run out in our laboratory and although a new batch was ordered months in advance, it did not arrive by the readout of our CUS experiments. In the case of CD44, as the first CRS experiments started to come out and we did not see significant changes in total CD4+ and Treg cells in blood, we sought the need to look at other cells populations, such as effector T cells, and this was incorporated in the spleen. Later, this marker was incorporated in blood and spleen for CUS mice.

Future studies need to evaluate different Treg cells doses and times of administration to determine their effects on depressive-like symptoms and analyze other brain regions for a more comprehensive understanding of the potential therapeutic effect that Treg cells could have on depressive and anxiety disorders. In addition, to delve deeper into the morphological changes observed in microglia, it would be necessary to consider subsequent 3D studies with thick sections. Moreover, we believe that it is necessary to see if similar effects are observed in female mice, which were not evaluated in this study.

## Data availability statement

The raw data supporting the conclusions of this article will be made available by the authors, without undue reservation.

## Ethics statement

The animal study was approved by the Ethics Committee of Universidad San Sebastian (internal code 03-2019-20) and Universidad de Concepción (internal code CEBB533-2019). The study was conducted in accordance with the local legislation and institutional requirements.

## Author contributions

YC: Formal analysis, Investigation, Methodology, Writing – original draft. RE-V: Conceptualization, Data curation, Formal analysis, Funding acquisition, Investigation, Methodology, Project administration, Resources, Software, Supervision, Validation, Visualization, Writing – original draft, Writing – review & editing. CG: Formal analysis, Investigation, Methodology, Writing – original draft. CT: Investigation, Methodology, Writing – original draft. MA: Investigation, Methodology, Writing – original draft. POl: Investigation, Methodology, Supervision, Writing – original draft. POr: Investigation, Methodology, Writing – original draft. CC: Investigation, Methodology, Supervision, Writing – review & editing. PV: Investigation, Methodology, Resources, Software, Supervision, Writing – review & editing. PL-C: Investigation, Software, Writing – review & editing. MG-R: Investigation, Resources, Writing – review & editing. KO: Writing – original draft, Writing – review & editing, Conceptualization, Data curation, Formal analysis, Funding acquisition, Investigation, Methodology, Project administration, Resources, Software, Supervision, Validation, Visualization.
